# Partial Replacement of Dietary Fat with Krill Oil or Coconut Oil Alleviates Dyslipidemia by Partly Modulating Lipid Metabolism in Lipopolysaccharide-Injected Rats on a High-Fat Diet

**DOI:** 10.3390/ijerph19020843

**Published:** 2022-01-12

**Authors:** Hee-Kyoung Son, Bok-Hee Kim, Jisu Lee, Seohyun Park, Chung-Bae Oh, Sunyoon Jung, Jennifer K. Lee, Jung-Heun Ha

**Affiliations:** 1 Research Center for Industrialization of Natural Neutralization, Dankook University, Cheonan 31116, Korea; kyoung1033@dankook.ac.kr (H.-K.S.); dlwltn970811@naver.com (J.L.); sb5590@naver.com (S.P.); syjung583@dankook.ac.kr (S.J.); 2 Department of Food and Nutrition, Chosun University, Gwangju 61452, Korea; kimbh@chosun.ac.kr; 3 Department of Food Science and Nutrition, Dankook University, Cheonan 31116, Korea; 4 Office of Technical Liaison, Industry Support Team, Gyeongnam Branch Institute, Korea Institute of Toxicology, Jinju 52834, Korea; cboh@kitox.re.kr; 5 Food Science and Human Nutrition Department, University of Florida, Gainesville, FL 32611, USA

**Keywords:** dyslipidemia, high-fat diet, lipopolysaccharide, krill oil, coconut oil

## Abstract

This study investigated the effects of partial replacement of dietary fat with krill oil (KO) or coconut oil (CO) on dyslipidemia and lipid metabolism in rats fed with a high-fat diet (HFD). Sprague Dawley rats were divided into three groups as follows: HFD, HFD + KO, and HFD + CO. The rats were fed each diet for 10 weeks and then intraperitoneally injected with phosphate-buffered saline (PBS) or lipopolysaccharide (LPS) (1 mg/kg). The KO- and CO-fed rats exhibited lower levels of serum lipids and aspartate aminotransferases than those of the HFD-fed rats. Rats fed with HFD + KO displayed significantly lower hepatic histological scores and hepatic triglyceride (TG) content than rats fed with HFD. The KO supplementation also downregulated the adipogenic gene expression in the liver. When treated with LPS, the HFD + KO and HFD + CO groups reduced the adipocyte size in the epididymal white adipose tissues (EAT) relative to the HFD group. These results suggest that KO and CO could improve lipid metabolism dysfunction.

## 1. Introduction

Over the decades, the prevalence of obesity has dramatically increased in developed and developing countries worldwide [1]. According to recent estimates by the World Health Organization (WHO), more than 1.9 billion adults aged 18 years and older were overweight, and of those, over 650 million were obese [2]. Moreover, the prevalence of obesity in the United States was reported to be 42.4% in 2017–2018 [3]. If the current trends continue, approximately one in five adults worldwide will be obese by 2025 [4]. Moreover, significant lifestyle changes due to the unexpected coronavirus disease 2019 (COVID-19) pandemic have resulted in a much higher incidence of obesity than expected [5]. Reduced physical activity, increased consumption of high calories (sugar and lipid) and convenience foods, and stress are some significant changes that may contribute to weight gain and obesity [6].

Obesity is caused by the accumulation of body fat when exposed to chronic positive energy balance due to excessive energy intake [7]. Obesity is considered a state of low-grade systemic inflammation that causes health complications, such as hyperglycemia, dyslipidemia, and hypertension [8], and increases the risk of chronic diseases, such as type 2 diabetes (T2D), nonalcoholic fatty liver (NAFLD), and cardiovascular disease (CVD) [9]. Current evidence indicates that lipids, particularly fatty acids (FAs), which comprise a major component of the adipose tissue (AT), play a significant role in the development of obesity and obesity-related metabolic pathogenesis [10,11]. Many studies have reported that the type of fatty acid consumed is more critical than the total fat intake [12,13]. The physiological and metabolic effects of fatty acids are determined by types of fatty acids such as saturated, monounsaturated, polyunsaturated, and trans-fatty acids [14]. WHO recommends not to exceed 30% from total energy intake [15]. Moreover, saturated fatty acids and trans-fatty acids are recommended not to exceed of 10% and 1%, respectively from total energy intake [16].

Krill oil (KO) is a rich source of omega-3 (*n*-3) polyunsaturated fatty acids (PUFAs) such as docosahexaenoic acid (DHA) and eicosapentaenoic acid (EPA) [17]. The proportion of *n*-3 PUFAs in KO is approximately 25% (*w*/*w*), where EPA and DHA comprise approximately 14% (*w*/*w*) and 6.5% (*w*/*w*), respectively [18]. *n*-3 PUFAs in KO exist in the form of phospholipids, which possess higher bioavailability and absorption rate compared to fish oil in the form of triacylglycerol [19,20]. KO also contains biologically active components, such as astaxanthin and vitamins A and E [21]. Previous studies have reported that these KO components have various physiological effects, such as anti-hyperlipidemic [22], anti-inflammatory [23], anti-oxidative stress [24], anti-arthritis [25], and neurocognitive improvement [26]. In particular, the anti-obesity effects have been demonstrated to reduce weight gain and improve serum lipid levels in several animal and clinical trials utilizing krill oil supplements [22,27,28,29,30,31,32,33,34]. KO suppressed the expression of proinflammatory mediators and cytokines by inhibiting the phosphorylation of p38 mitogen-activated protein kinase (MAPK) and c-Jun N-terminal kinase (JNK) in lipopolysaccharide (LPS)-stimulated RAW 264.7 macrophages [23].

Medium-chain fatty acids (MCFAs) are fatty acids consisting of 6–12 carbon atoms, and oils such as coconut oil and palm kernel oil are significantly enriched with MCFAs (approximately 55% of the total fat composition) [35]. Coconut oil (CO) is an edible oil that has gained popularity in recent years and is mainly composed of lauric acid, a medium-chain fatty acid [36,37]. As absorbed in the small intestine and directly transported to the liver by the portal blood system, MCFAs are quickly used as an energy source in the body [38,39]. Dietary MCFAs have been reported to enhance thermogenesis, inhibit fat deposition through fat oxidation, and preserve insulin sensitivity [40]. On the other hand, some studies have claimed that in a typical Western diet, coconut oil consumption causes acute inflammation similar to other SFAs sources [41]. To date, controversy remains regarding the nutritional values and functions of CO.

Administration of LPS, a major component of the outer membrane of Gram-negative bacteria, leads to a rapid and transitory increase in the levels of proinflammatory cytokines such as tumor necrosis factor alpha (TNF-α) and interleukin (IL)-6 in mammals [42]. As a result, it induces acute inflammation in multiple organs, including the kidney, brain, lung, and liver [43]. Thus, several studies have investigated the anti-inflammatory effects of natural products, compounds, or drugs using LPS-induced animals as models of acute inflammation [44,45,46,47]. Recently, we reported that the partial replacement of SFAs in a high-fat diet (HFD) with PUFAs such as perilla oil (PO) or corn oil at a converted dose (2–8%) suppressed insulin resistance and nuclear factor κ-light-chain-enhancer of activated B cells (NF-κB) signaling pathways, and enhanced antioxidant enzyme expression in rats [48]. Intriguingly, we observed that PO and corn oil exacerbated dyslipidemia by elevating triglyceride (TG), total cholesterol (TC), non-high-density lipoprotein-cholesterol (non-HDL-C), aspartate aminotransferase (AST), alanine aminotransferase (ALT), and alkaline phosphatase (ALP) levels when injected with LPS. As *n*-3 PUFAs are highly prone to oxidation due to their chemical structures, we postulated that these effects might be attributed to oxidative damage and reduced α-tocopherol from *n*-3 PUFAs consumption [49,50]. To compensate for these adverse effects of *n*-3 PUFAs previously reported, in this study, we increased the dose of *n*-3 PUFAs in KO by 5%. As a comparison, HFD was replaced with 5% MCFAs, as CO was also included in this study. In this study, we investigated the effects of partial replacement of dietary fat with KO and CO on metabolic changes induced by LPS administration in HFD-fed rats.

## 2. Materials and Methods

### 2.1. Animal Experiments and Diets

All animal studies were approved by the Dankook University Institutional Animal Care and Use Committee (IACUC, No. DKU-21-051). Five-week-old male Sprague Dawley rats were obtained from the DooYeol Biotech (Seoul, Korea). The KO was obtained from Biocorp (Goheung-gun, Jeollanam-do, Korea), and the CO (Wellspring extra virgin coconut oil, Peter Paul Candy Manufacturing Company, Manila, Philippines) was purchased from a local market in South Korea. A total of 48 rats were housed under a controlled temperature of 22 ± 1 °C and humidity of 55 ± 5% with a 12 h light/12 h dark cycle. The experimental rats were randomly assigned to one of the three dietary groups (n = 16 per group): (1) high-fat diet (HFD); (2) HFD partially replaced with 5% krill oil (*w*/*w*) (HFD + KO); and (3) HFD partially replaced with 5% coconut oil (*w*/*w*) (HFD + CO). The dietary composition of HFD, HFD + KO, and HFD + CO is listed in Table 1. The animals had ad libitum access to the diet and purified water. Following 10 weeks of experimental diet consumption, each group was further divided into two subgroups: (1) an intraperitoneal injection of LPS (1 mg/kg body weight); or (2) phosphate-buffered saline (PBS) (1 mL/kg body weight). After 24 h of PBS or LPS injection, the experimental rats were sacrificed by thoracotomy after CO_2_ narcosis. Blood was collected by cardiac puncture and subsequently centrifuged at 3000× *g* at 4 °C for 15 min to isolate the serum. The liver and adipose tissues were harvested and weighed after washing with normal saline solution. The tissues and serum samples were stored at −80 °C until further analysis.

### 2.2. Analysis of the Fatty Acid Composition of Experimental Diets

The fatty acid composition of the experimental diets was determined by methyl esterification of boron trifluoride (BF_3_)-methanol [48]. Briefly, 0.1 g of sample was placed in a test tube, followed by the addition of 0.5 mL of heptadecanoic acid (C17:0; 1 mg/mL hexane). NaOH–methanol (2 mL 0.5 N NaOH-methanol) was added to the mixture and heated at 110 °C for 10 min. After cooling to room temperature, 4 mL of BF_3_-methanol was added to the mixtures and reheated at 110 °C for 1 h. Thereafter, 2 mL of hexane was added to the mixture and vortexed for 1 min to collect the hexane layer for lipid analysis. Fatty acids were analyzed using gas chromatography (GC) (Agilent Technologies 6890N, Agilent Technologies, CA, USA). Fatty acids were identified by comparing the peak retention times of those of the reference standard solution Supelco 37-component FAME mix (Sigma-Aldrich Co., St. Louis, MO, USA) and then expressed as a percentage of the total analyzed fatty acids. The fatty acid composition of the experimental diets is shown in Table 2.

### 2.3. Oral Glucose Tolerance Test (OGTT) and Insulin Tolerance Test (ITT)

The oral glucose tolerance test (OGTT) was performed in experimental rats during the 6th week of the dietary intervention. Following a 12 h fast, blood samples were collected by the lateral tail vein incision to determine fasting glucose levels. The rats were then gavaged with a glucose solution (1 g/kg body weight). Blood glucose level was then analyzed at 0, 30, 60, 90, and 120 min after glucose ingestion. Glucose levels were measured using Accu-Chek Instant Test Strips (Accu-Chek, Seoul, Korea) and an Accu-Chek Instant blood glucose meter. The insulin tolerance test (ITT) was conducted at the end (10th week of feeding) of the study. Overnight fasted rats were intraperitoneally injected with insulin at 1 U/kg body weight. Blood glucose levels were measured at 0, 15, 30, 60, 120, and 210 min during the ITT. Furthermore, glucose and insulin tolerance were evaluated by calculating the area under the curve (AUC) for serum glucose and insulin levels.

### 2.4. Analysis of Fatty Acids Composition of Whole Blood

A drop of whole blood was collected on a blood spot card pretreated with a multicomponent antioxidant cocktail. The blood spot card was purchased from the OmegaQuant Analytics (Sioux Falls, SD, USA), and the blood fatty acid composition was analyzed using GC, as described by Harris and Polreis [51]. The fatty acid composition of whole blood was expressed as a percentage of the total analyzed fatty acids.

### 2.5. Determination of Serum Metabolic Parameters

Serum levels of AST, ALT, ALP, TG, TC, and HDL-C were measured using commercially available kits (MBL, Gunpo, Korea). The non-HDL-C level was calculated by subtracting the HDL-C from the TC. The atherogenic coefficient (AC) was calculated as ((TC − HDL-C) /HDL-C), and cardiac risk factor (CRF) was determined using the following established formula: (TC/HDL-C) [52]. Serum glucose (Crystal Chem, Downers Grove, IL, USA) and insulin (Mercodia AB, Uppsala, Sweden) levels were analyzed using commercial kits according to the manufacturer’s instructions. The homeostatic index of insulin resistance (HOMA-IR) values was calculated using the following formula: HOMA-IR = fasting insulin (μU/mL) × fasting glucose (mmol/L)/22.5 [53].

### 2.6. Measurement of Serum IL-1β and MCP-1 Levels

The serum levels of IL-1β and monocyte chemoattractant protein-1 (MCP-1) were evaluated using a MILLIPLEX map Luminex assay kit (Millipore, Danvers, MA, USA), according to the manufacturer’s protocol.

### 2.7. Measurement of Lipid Contents in the Liver

Lipids were extracted from the liver using the method of Bligh and Dyer [54] with slight modifications [48]. Briefly, 0.1 g of tissue was added to a chloroform/methanol (1:2, *v*/*v*) solution and centrifuged at 1800× *g* for 5 min to obtain the lower lipid layer containing TG and TC for analysis. Subsequently, the TG and TC contents were determined using commercial kits (MBL, Gunpo, Korea).

### 2.8. Histological Assessment of the Liver and Epididymal Adipose Tissue

A portion of the liver and adipose tissue was fixed with a 10-fold volume of 10% formalin. Thereafter, the tissues were embedded in paraffin blocks and sectioned using a cryo-cut microtome (Leica CM1800, Wetzler, Germany) with a thickness of 3–4 μm. The sections were stained with hematoxylin and eosin (H&E) and captured using an optical microscope (ZEISS Axio Imager 2, Carl Zeiss, Oberkochen, Germany). The liver histological changes were assessed (by a blinded observer) in three different randomly selected 20X fields for each experimental rat. Histological scores were measured using Kleiner’s histological scoring system [55] by quantifying the degree of inflammatory cell infiltration, steatosis, and balloon cells. Adipocyte size (μm^2^) and the number of crown-like structures (CLS) were determined in three different randomly selected 20X fields for each experimental rat. Adipocyte size and CLS number were measured using an Image J (NIH, Bethesda, MD, USA).

### 2.9. Quantitative Real-Time Polymerase Chain Reaction (qRT-PCR)

Total RNA was isolated from liver and epididymal adipose tissue using the NucleoZoL reagent (Macherey-Nagel, GmbH & Co. KG, Düren, Germany). The complementary-DNA (cDNA) was synthesized from 1 μg of messenger ribonucleic acid (mRNA) using the iScript™ cDNA Synthesis Kit (Bio-Rad Laboratories, CA, USA). The quantitative polymerase chain reaction (qPCR) was performed using the CFX96 Real-Time PCR Detection System (Bio-Rad Laboratories) and conducted using iQ™ SYBR^®^ Green Supermix (Bio-Rad Laboratories). Quantitative PCR data were evaluated using the comparative critical threshold (Ct) method and were normalized using GAPDH as an internal control (primer sequences are shown in Table 3). Relative gene expression was calculated using the ΔΔCt method and expressed as a fold change in the HFD group (PBS group).

### 2.10. Statistical Analysis

Data were presented as means and standard deviations (SDs) or box-and-whisker plots. Two-way analysis of variance (ANOVA) was used to determine the main effects of diet and LPS stimulation or their interactions. Tukey’s multiple comparison test was performed when there was a significant interaction between two factors (*p* < 0.05). A summary of the two-way ANOVA results from the main and interaction effects is provided in Table 4. The one-way ANOVA was used to compare the means of the three groups. Differences were considered statistically significant at *p* < 0.05. All statistical analyses were performed using XLSTAT 2012 for windows (Addinsoft Inc., Paris, France).

## 3. Results

### 3.1. Effect of the Dietary Fat Replacement with KO or CO on Body Weight Changes, Food Intake, Energy Intake, and Food Efficiency Ratio (FER)

**Figure 1:**
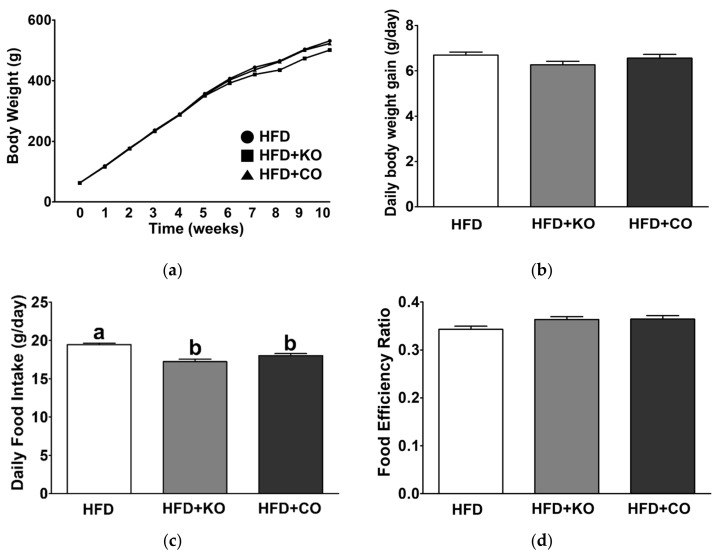
Effects of partial replacement of dietary fat with krill oil or coconut oil on the body weight, daily body weight gain, food intake, and food efficiency ratio in HFD-fed rats. Sprague Dawley rats were fed either a high-fat diet (HFD; open box), HFD partially replaced with krill oil (HFD + KO; gray-filled box), or coconut oil (HFD + CO; black-filled box) for 10 weeks. (**a**) Body weight changes, (**b**) daily body weight gain, (**c**) daily food intake, and (**d**) food efficiency ratio (FER) were measured. Values are presented as means ± SD; n = 16 per individual group. Data were analyzed using one-way ANOVA followed by Tukey’s multiple comparisons test; labeled means without a common letter differ significantly, *p* < 0.05. HFD, high-fat diet; HFD + KO, high-fat diet + krill oil; HFD + CO, high-fat diet + coconut oil.

During the experimental period, body weight (BW) did not vary according to dietary intervention (Figure 1a). After seven weeks of experimental feeding, rats fed with HFD + KO diet displayed relatively lower BW; however, BW was not different at other weeks (*p* = 0.058~0.194). Thus, the daily BW gain is not altered by the various diets (Figure 1b). Intriguingly, the daily food intake of experimental rats fed with HFD + KO or HFD + CO diet is significantly lower than that of HFD-fed rats (Figure 1c). HFD generally induces excess dietary intake, but dietary fat replacement with PUFAs may decrease overconsuming energy [56]. Due to lower energy intake, HFD + KO- and HFD + CO-fed rats showed elevated FER compared to HFD-fed rats, whereas the difference was not statistically significant (Figure 1d).

### 3.2. Effect of the Dietary Fat Replacement with KO or CO on Liver and Adipose Tissue Weights

**Figure 2:**
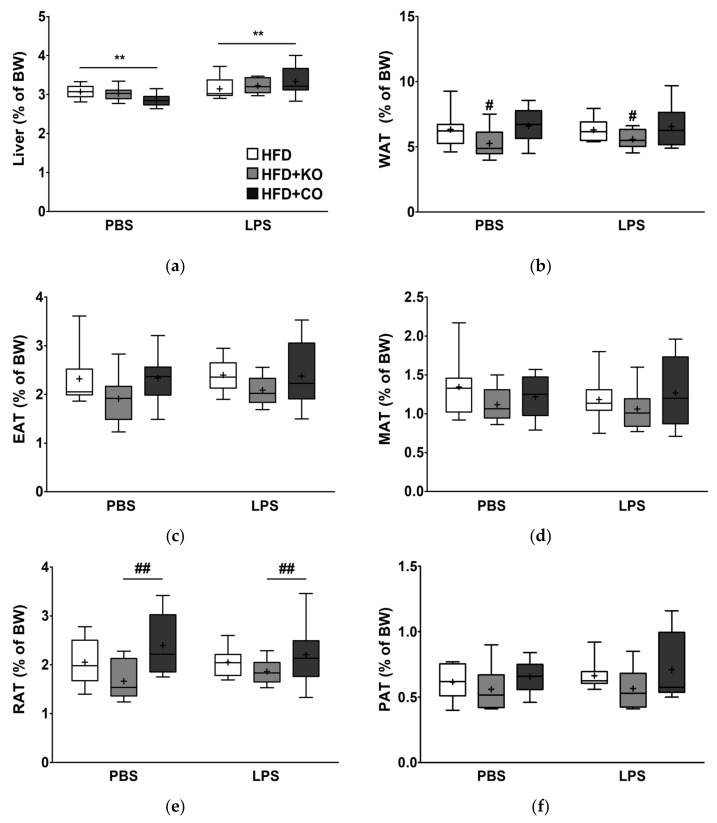
Effects of partial replacement of dietary fat with krill oil or coconut oil on hepatomegaly and adiposity in rats after the LPS challenge. Sprague Dawley rats were fed either a high-fat diet (HFD), HFD partially replaced with krill oil (HFD + KO), or coconut oil (HFD + CO) for 10 weeks, and then treated with PBS or LPS (1 mg/kg) for 24 h. (**a**) Liver, (**b**) total white adipose tissue (WAT), (**c**) epididymal white adipose tissue (EAT), (**d**) mesenteric white adipose tissue (MAT), (**e**) retroperitoneal white adipose tissue (RAT), and (**f**) perirenal white adipose tissue (PAT) weights were measured. Values are displayed as box-and-whisker plots; n = 8 per individual group. Data were analyzed by two-way ANOVA followed by Tukey’s multiple comparisons test to determine the interactions or the main effects (diet and LPS stimulation). Asterisk indicates a significant main effect for LPS (** *p* < 0.01). Pound indicates a significant main effect for diet (^#^ *p* < 0.05, ^##^ *p* < 0.01). The mean values are indicated by “+” signs. HFD, high-fat diet; HFD + KO, high-fat diet + krill oil; HFD + CO, high-fat diet + coconut oil; PBS, phosphate-buffered saline; LPS, lipopolysaccharide; BW, body weight.

To determine whether the partial replacement of fat with KO or CO affects the weight of the metabolic organs following LPS challenge, the relative weights of the liver, visceral fat depot, and total WAT (WAT; as the sum of the weight of epididymal (EAT), mesenteric (MAT), retroperitoneal (RAT), and perirenal adipose tissue (PAT)) were measured and expressed as a percentage of BW. In the liver, the LPS-treated group showed a significant weight increase (1.1-fold increase; *p* < 0.01) compared to the PBS-treated group (Figure 2a). Within the PBS-treated group, no differences were observed among the different subgroups; however, rats fed with HFD + KO tended to exhibit lower liver weights compared to those of HFD-fed rats. When stimulated with LPS, the weight of the liver of the different subgroups did not exhibit significant differences. The adipose tissue weights tended to be lower in the HFD + KO group than in the HFD and HFD + CO groups, regardless of the LPS treatment (Figure 2b–f). Furthermore, significant main effects of diets on weights of WAT and RAT using two-way ANOVA were noted in rats fed HFD + KO diets, *p* < 0.05, *p* < 0.01, respectively. In summary, these data showed that LPS treatment caused hepatomegaly, disregarding the dietary intervention, and the HFD + KO diet may be associated with decreased WAT accumulation.

### 3.3. Effect of the Dietary Fat Replacement with KO or CO on Serum Lipid Profiles and Cardiovascular Parameters

**Figure 3:**
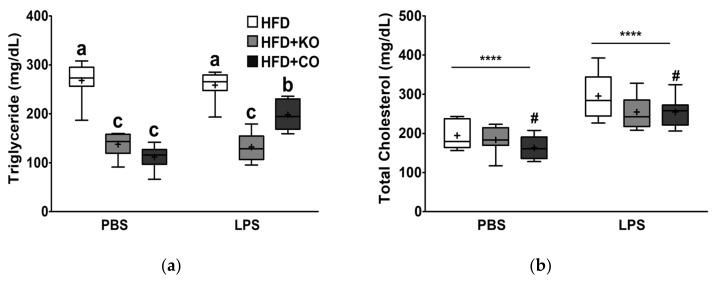

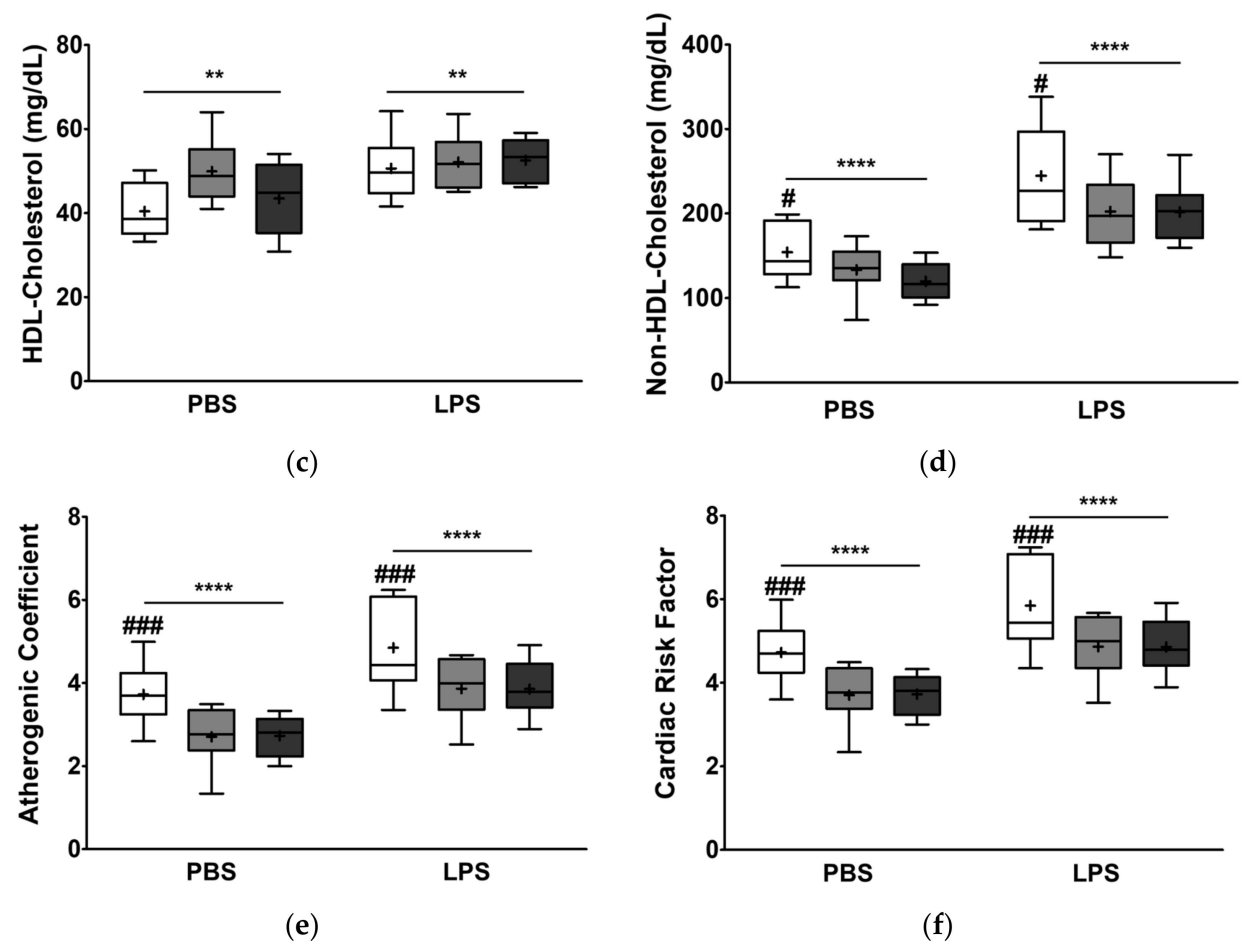
Effects of partial replacement of dietary fat with krill oil or coconut oil on serum lipid profiles and cardiovascular indices in rats after the LPS challenge. Sprague Dawley rats were fed either a high-fat diet (HFD), HFD partially replaced with krill oil (HFD + KO), or coconut oil (HFD + CO) for 10 weeks, and then treated with PBS or LPS (1 mg/kg) for 24 h. (**a**) Serum triglyceride, (**b**) total cholesterol, (**c**) high-density lipoprotein (HDL)-cholesterol, (**d**) non-HDL cholesterol levels, (**e**) atherogenic coefficient (AC), and (**f**) cardiac risk factor (CRF) were measured. Values are displayed as box-and-whisker plots; n = 8 per individual group. Data were analyzed by two-way ANOVA followed by Tukey’s multiple comparisons test to determine the interactions or the main effects (diet and LPS stimulation. Asterisk indicates a significant main effect for LPS (** *p* < 0.01, **** *p* < 0.0001). Pound indicates a significant main effect for diet (^#^ *p* < 0.05, ^###^ *p* < 0.001). Labeled means without a common letter differ significantly, *p* < 0.05. The mean values are indicated by “+” signs. HFD, high-fat diet; HFD + KO, high-fat diet + krill oil; HFD + CO, high-fat diet + coconut oil; PBS, phosphate-buffered saline; LPS, lipopolysaccharide.

To investigate the effects of partial substitution of fat for either KO or CO on CVD prevention and lipid-lowering, we measured the levels of CVD-related serum lipid profiles. After LPS injection, the serum TG, TC, HDL-C, non-HDL-C, AC, and CRF levels remarkably increased by 1.14-, 1.49-, 1.16-, 1.59-, 1.37-, and 1.28-fold, respectively (Figure 3a–f). A significant interaction between LPS and the experimental diet was observed (interaction, *p* < 0.0001), in which feeding of KO or CO remarkably decreased the serum TG levels both in the presence and absence of LPS (Figure 3a). In the vehicle group, serum HDL-C levels were higher in the HFD + KO-or HFD + CO-fed rats than in the HFD-fed rats, whereas the levels in the LPS treatment group were similar among the different dietary groups (Figure 3c). The consumption of HFD + KO or HFD + CO decreased serum non-HDL-C levels compared to the HFD group, regardless of LPS stimulation (Figure 3d). Thus, KO and CO significantly inhibited the HFD-induced (*p* < 0.0001) and LPS-stimulated (*p* < 0.001) CVD expectancies (atherogenic coefficient and cardiac risk factor) (Figure 3e,f). Cumulatively, these observations indicated that fat replacement with KO or CO improved the serum lipid profiles; in particular, KO had a positive effect on CVD prevention and lipid-lowering, regardless of the LPS challenge.

### 3.4. Effect of the Dietary Fat Replacement with KO or CO on Oral Glucose Tolerance (OGTT) and Insulin Tolerance Test (ITT)

**Figure 4:**
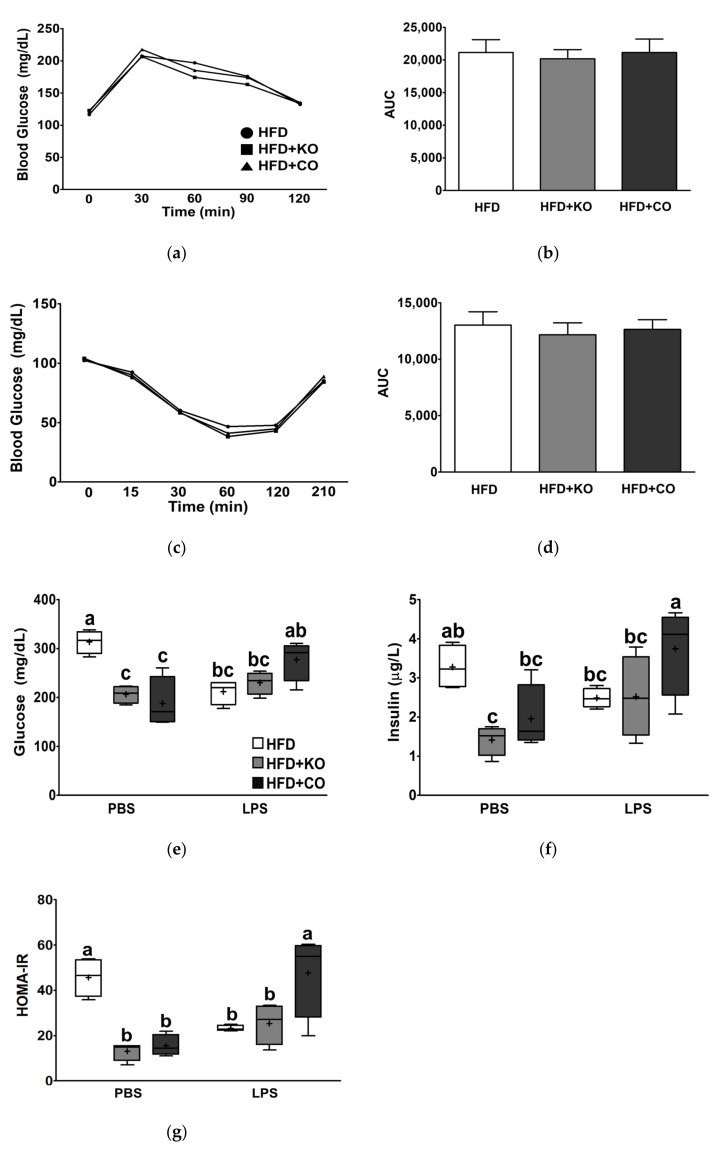
Effects of partial replacement of dietary fat with krill oil or coconut oil on glucose metabolism in rats. Sprague Dawley rats were fed either a high-fat diet (HFD), HFD partially replaced with krill oil (HFD + KO), or coconut oil (HFD + CO) for 10 weeks. (**a**) Oral glucose tolerance test (OGTT), n = 16, (**b**) the area under the curve (AUC) during OGTT, (**c**) insulin tolerance test (ITT), n = 16, (**d**) AUC during ITT, (**e**) serum glucose level, n = 8, (**f**) serum insulin level, n = 8, and (**g**) homeostasis model assessment of insulin resistance (HOMA-IR), n = 8, were assessed. Data were analyzed by one-way or two-way ANOVA followed by Tukey’s multiple comparisons test to determine the interactions or the main effects (diet and LPS stimulation). Labeled means without a common letter differ significantly, *p* < 0.05. The mean values are indicated by “+” signs. HFD, high-fat diet; HFD + KO, high-fat diet + krill oil; HFD + CO, high-fat diet + coconut oil; PBS, phosphate-buffered saline; LPS, lipopolysaccharide.

During the 6th week of the dietary intervention, OGTT and ITT were performed to determine whether animals consuming HFD + KO or HFD + CO improved glucose utilization, in which FER was increased. Regardless of dietary interventions, blood glucose reached the apex of 30 min after the glucose challenge; however, HFD + KO cleared blood glucose relatively faster than other experimental groups at 30 min (Figure 4a). Blood glucose levels were not significantly different between the experimental groups at 60 and 120 min. ITT was conducted to understand whether high-fat replacement with KO or CO might alter insulin resistance (Figure 4c). However, the fat replacement did not change blood glucose levels after insulin injection at 1 mg/kg BW for 120 min. The calculated AUC values in the OGTT and ITT did not differ significantly among the experimental diets (Figure 4b,d). Together, these results indicate that the replacement of fat with KO or CO did not affect glucose tolerance or insulin resistance.

### 3.5. Effect of the Dietary Fat Replacement with KO or CO on Serum Glucose and Insulin Levels

As KO or CO consumption remarkably improved HFD-induced and LPS-stimulated serum metabolic parameters, we investigated whether KO or CO could also alter insulin, glucose, and HOMA-IR in serum at the end of the dietary intervention. LPS treatment slightly increased serum glucose and insulin levels (only insulin, *p* < 0.05; Figure 4e,f). Serum glucose and insulin levels in rats fed with HFD + KO diet in the absence of LPS were significantly lower than those in the HFD group (*p* < 0.05), but they did not decrease in the presence of LPS. Partial replacement of HFD with KO or CO significantly lowered the HOMA-IR index in the PBS group, whereas HFD + CO showed higher levels than those of HFD in the LPS-stimulated group (interaction, *p* < 0.0001; Figure 4g).

### 3.6. Effect of the Dietary Fat Replacement with KO or CO on Blood Fatty Acid Composition

Table 5 shows the changes in fatty acid composition detected in the whole blood of rats. The contents of SFAs and MUFAs in whole blood were not affected by the LPS and dietary interventions. The HFD + KO diet markedly increased the levels of major *n*-3 PUFAs (i.e., EPA, DPA, and DHA), regardless of the LPS challenge (*p* < 0.001). Conversely, rats in the HFD + CO group markedly increased the levels of arachidonic acid (AA), docosatetraenoic acid, docosapentaenoic acid, and total *n*-6 PUFAs (*p* < 0.001). Consequently, the *n*-6/*n*-3 and AA/EPA ratio, a marker of inflammation, were significantly higher in the HFD + CO group than in the other groups, whereas rats fed with HFD + KO diet showed a significant reduction (*p* < 0.0001).

### 3.7. Effect of the Dietary Fat Replacement with KO or CO on Serum Proinflammatory Cytokines, and Chemokine Levels

**Figure 5:**
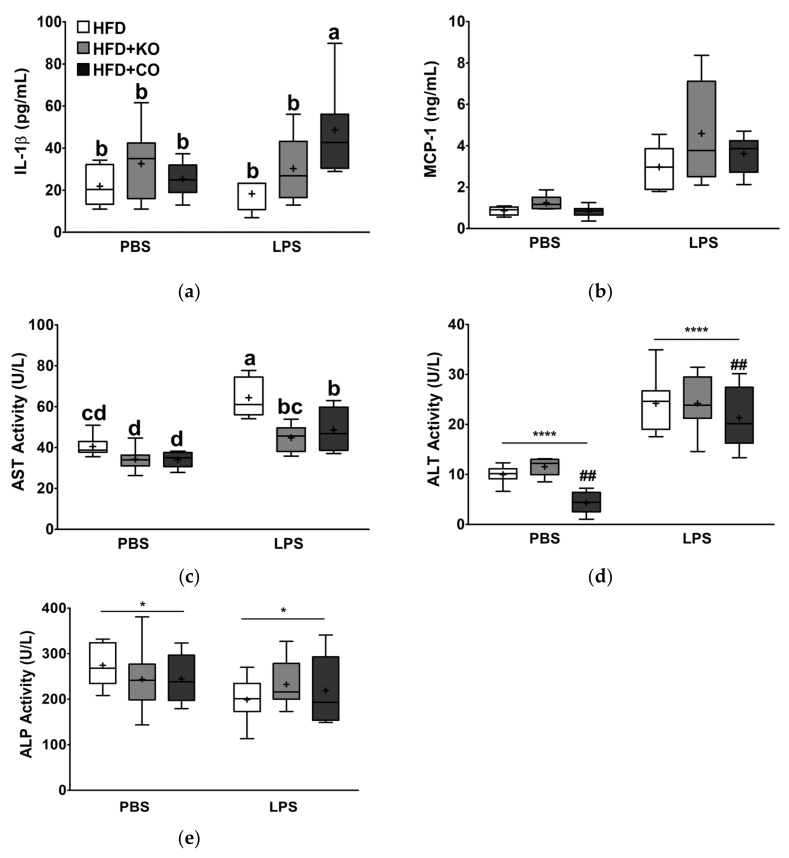
Effects of partial replacement of dietary fat with krill oil or coconut oil on serum proinflammatory cytokines, chemokine, and hepatic function enzyme levels in rats after the LPS challenge. Sprague Dawley rats were fed either a high-fat diet (HFD), HFD partially replaced with krill oil (HFD + KO), or coconut oil (HFD + CO) for 10 weeks, and then treated with PBS or LPS (1 mg/kg) for 24 h. (**a**) Interleukin (IL)-1β levels, (**b**) monocyte chemoattractant protein (MCP)-1 levels, (**c**) aspartate aminotransferase (AST), (**d**) alanine aminotransferase (ALT), and (**e**) alkaline phosphatase (ALP) activities were analyzed. Values are displayed as box-and-whisker plots, n = 8 per individual group. Data were analyzed by two-way ANOVA followed by Tukey’s multiple comparisons test to determine the interactions or the main effects (diet and LPS stimulation). Asterisk indicates a significant main effect for LPS (* *p* < 0.05, **** *p* < 0.0001). Pound indicates a significant main effect for diet (^##^ *p* < 0.01). Labeled means without a common letter differ significantly, *p* < 0.05. The mean values are indicated by “+” signs. HFD, high-fat diet; HFD + KO, high-fat diet + krill oil; HFD + CO, high-fat diet + coconut oil; PBS, phosphate-buffered saline; LPS, lipopolysaccharide.

To explore the effects of fat replacement with KO or CO on proinflammatory status, the serum levels of cytokines and chemokines, such as IL-1β and MCP-1, were evaluated. Although LPS stimulation tended to increase IL-1β but did not reach statistical significance (*p* = 0.219), the MCP-1 levels significantly increased by 3.83-fold (*p* < 0.0001; Figure 5a,b). The IL-1β levels in rats fed with HFD + CO following LPS stimulation were significantly higher than those in other groups (interaction, *p* < 0.05). Interestingly, in the LPS-stimulated group, higher MCP-1 levels were observed in the HFD + KO group than in the HFD group, but the difference was not significant.

### 3.8. Effect of the Dietary Fat Replacement with KO or CO on Enzyme Profiles Related to Hepatic Function

To investigate the effects of partial replacement of fat with KO or CO on hepatic damage, we measured the activity of enzyme markers (ALT, AST, and ALP) of hepatic function following the LPS challenge. Changes in hepatic function enzymes in the serum are shown in Figure 5c–e. Compared to the PBS group, LPS injection markedly enhanced serum AST and ALT activities by 1.45-fold and 2.69-fold, respectively (*p* < 0.0001), but significantly decreased ALP activity. Serum AST activity was significantly lower in KO- or CO-fed rats in the PBS group than in rats fed with HFD + CO with LPS stimulation. Serum ALT activity was similar between the HFD and HFD + KO groups, whereas the HFD + CO group had lower activity than the other groups. Serum ALP activity of rats fed diets supplemented with KO or CO in the PBS group tended to be lower than that in the HFD group. Although the LPS group tended to increase serum ALP activity by KO or CO feeding, the dietary intervention did not affect serum ALP activity.

### 3.9. Effect of the Dietary Fat Replacement with KO or CO on Hepatotoxicity and Hepatic Fibrosis

**Figure 6:**
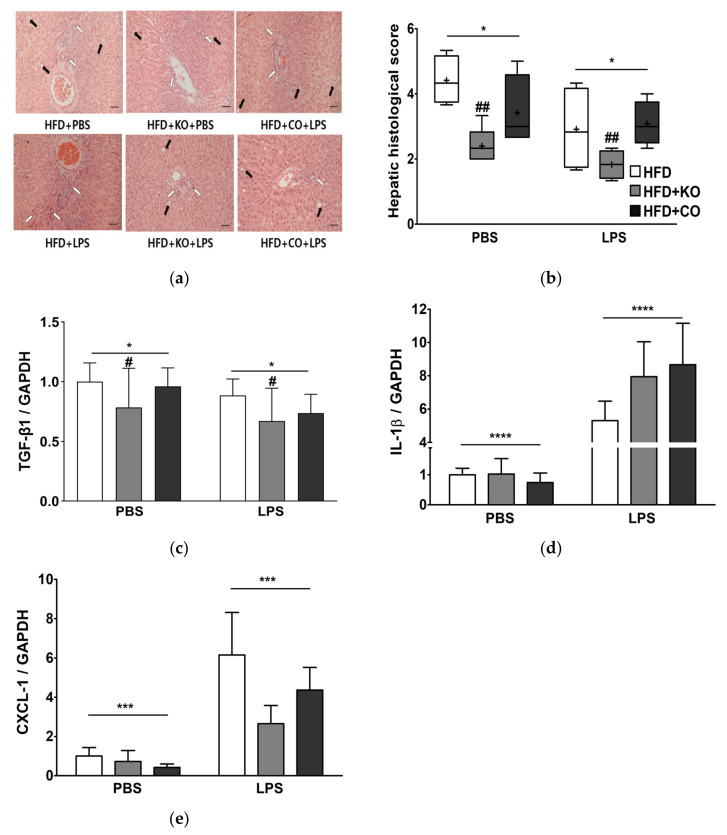
Effects of partial replacement of dietary fat with krill oil or coconut oil on the hepatic histopathology, and mRNA expression of proinflammatory genes in rats after the LPS challenge. Sprague Dawley rats were fed either a high-fat diet (HFD), HFD partially replaced with krill oil (HFD + KO), or coconut oil (HFD + CO) for 10 weeks, and then treated with PBS or LPS (1 mg/kg) for 24 h. (**a**) Hematoxylin and eosin (H&E) staining of liver tissue (bar = 50 μm, 20× magnification); black arrow indicates lipid accumulation and white arrow indicates inflammatory foci. (**b**) hepatic histological score, (**c**) transforming growth factor-β1 (TGF-β1), (**d**) interleukin 1 beta (IL-1β) level, and (**e**) chemokine (C-X-C motif) ligand 1 (CXCL-1) mRNA expression levels were assessed. Values are displayed as box-and-whisker plots, n = 8 per individual group. Data were analyzed by two-way ANOVA followed by Tukey’s multiple comparisons test to determine the interactions or the main effects (diet and LPS stimulation). Asterisk indicates a significant main effect for LPS (* *p* < 0.05, *** *p* < 0.001, **** *p* < 0.0001). Pound indicates a significant main effect for diet (^#^ *p* < 0.05, ^##^ *p* < 0.01). The mean values are indicated by “+” signs. HFD, high-fat diet; HFD + KO, high-fat diet + krill oil; HFD + CO, high-fat diet + coconut oil; PBS, phosphate-buffered saline; LPS, lipopolysaccharide.

Rats fed with HFD displayed degenerative changes in the hepatocytes with inflammatory cell infiltration and ballooning cell necrosis around the central vein area compared to the HFD + KO or HFD + CO groups (Figure 6a). Interestingly, the histological changes in the HFD + KO-supplemented rats were remarkably attenuated regardless of the LPS challenge. Consequently, inflammatory cells, steatosis, and hepatocellular ballooning in the liver were assessed and graded numerically (Figure 6b). The liver histological score was 42.27% and 11.36%, which was lower in rats fed with HFD or HFD + CO diet, respectively, compared to the HFD group (*p* < 0.01). These data suggest that fatty acid replacement with KO or CO resulted in reduced hepatic damage with improved liver function and lipid accumulation, regardless of the LPS challenge. The fibrosis-marker TGF-β1 expression was significantly decreased (*p* < 0.05) in the presence of LPS. Furthermore, the HFD + KO-fed rats showed lower TGF-β1 expression than the other experimental groups (*p* < 0.05, Figure 6c). The IL-1β and C-X-C motif chemokine ligand 1 (CXCL-1) levels in the liver were significantly upregulated by 7.88-fold and 6.10-fold, respectively, following LPS stimulation (Figure 6d,e) (*p* < 0.0001; IL-1β, *p* < 0.01; CXCL-1). The expression of CXCL-1 in the liver of rats fed with HFD + KO or HFD + CO in the LPS group tended to be lower than that in the HFD group.

### 3.10. Effect of the Dietary Fat Replacement with KO or CO on Fat Accumulation and mRNA Expression of Lipogenesis-related Genes in the Liver

**Figure 7:**
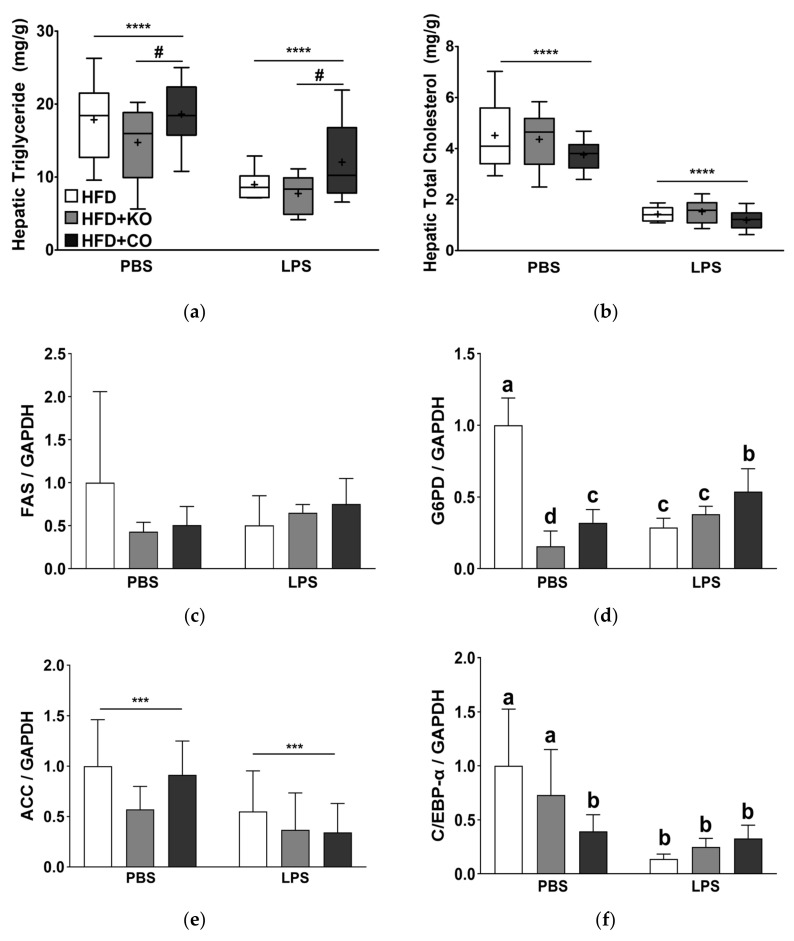

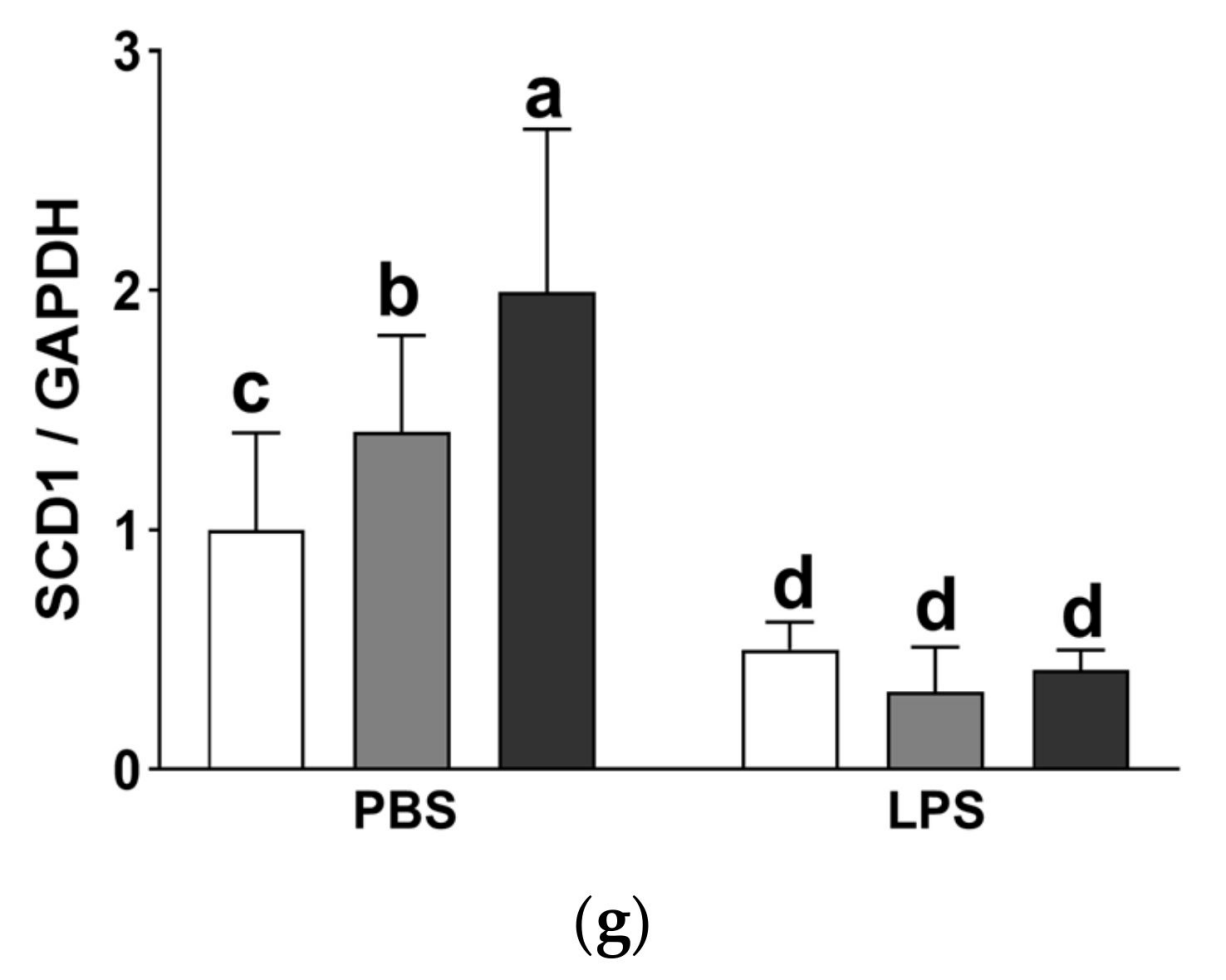
Effects of partial replacement of dietary fat with krill oil or coconut oil on the hepatic lipid contents and mRNA expression of lipogenesis-related genes in rats after the LPS challenge. Sprague Dawley rats were fed either a high-fat diet (HFD), HFD partially replaced with krill oil (HFD + KO), or coconut oil (HFD + CO) for 10 weeks, and then treated with PBS or LPS (1 mg/kg) for 24 h. (**a**) Hepatic triglyceride content, (**b**) hepatic total cholesterol content, (**c**) fatty acid synthase (FAS), (**d**) glucose-6-phosphate dehydrogenase (G6PD), (**e**) acetyl CoA carboxylase (ACC), (**f**) CCAAT/enhancer-binding protein alpha (C/EBP-α), and (**g**) stearoyl-CoA desaturase-1 (SCD1) mRNA expression levels were measured. Values are presented as means ± SD; n = 8 per individual group. Data were analyzed by two-way ANOVA followed by Tukey’s multiple comparisons test to determine the interactions or the main effects (diet and LPS stimulation). Asterisk indicates a significant main effect for LPS (*** *p* < 0.001, **** *p* < 0.0001). Pound indicates a significant main effect for LPS (^#^ *p* < 0.05). Labeled means without a common letter differ significantly, *p* < 0.05. HFD, high-fat diet; HFD + KO, high-fat diet + krill oil; HFD + CO, high-fat diet + coconut oil; PBS, phosphate-buffered saline; LPS, lipopolysaccharide.

Moreover, we measured the hepatic lipid levels, as shown in Figure 7a,b. Hepatic TG and TC levels were significantly reduced following the LPS challenge (*p* < 0.0001). Rats fed with HFD + KO had substantially lower hepatic TG levels than those in the HFD and HFD + CO groups (*p* < 0.05). However, hepatic TC levels did not vary considerably with dietary intervention (Figure 7b). LPS challenge decreased the hepatic glucose-6-phosphate dehydrogenase (G6PD), acetyl-CoA carboxylase (ACC), CCAAT enhancer-binding protein α (C/EBP-α), and stearoyl-CoA desaturase-1 (SCD1) mRNA expression by 18.24%, 49.19%, 66.04%, and 71.59%, respectively (Figure 7d–g). Prior to LPS treatment, fatty acid synthesis (FAS), G6PD, and ACC mRNA expression was decreased in the HFD + KO and HFD + CO groups compared to the HFD group (Figure 7c–e). However, following LPS treatment, FAS, C/EBP-α, and SCD1 mRNA expression were not altered by the HFD + KO and HFD + CO diets (Figure 7c,f,g).

### 3.11. Effect of the Dietary Fat Replacement with KO or CO on Hypertrophy and Macrophage Infiltration in Epididymal Adipose Tissue

**Figure 8:**
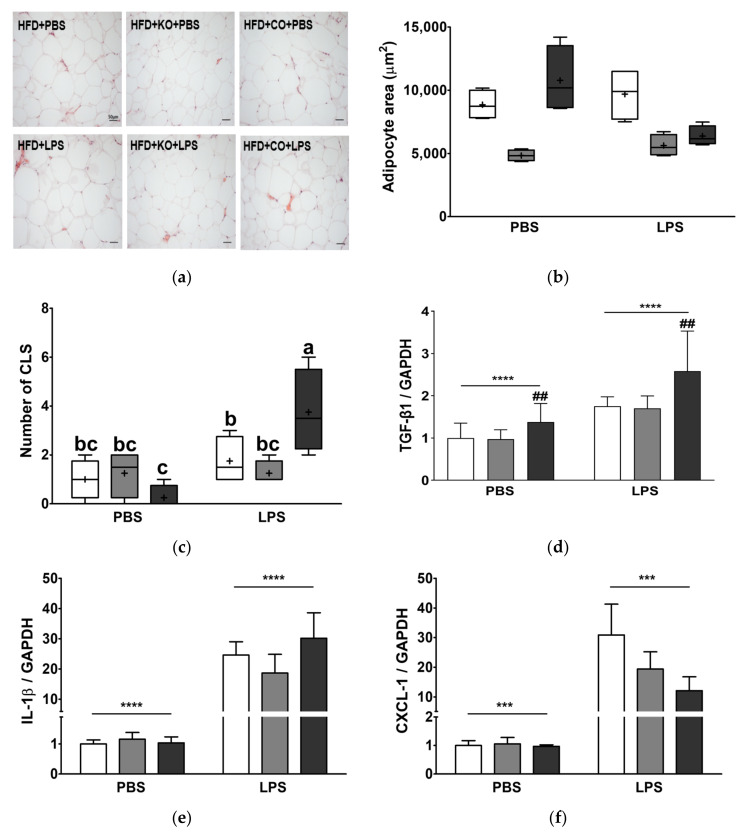
Effects of partial replacement of dietary fat with krill oil or coconut oil on the adipocyte histopathology and mRNA expression of proinflammatory genes in epididymal adipose tissues (EAT) of rats after the LPS challenge. Sprague Dawley rats were fed either a high-fat diet (HFD), HFD partially replaced with krill oil (HFD + KO), or coconut oil (HFD + CO) for 10 weeks, and then treated with PBS or LPS (1 mg/kg) for 24 h. (**a**) Hematoxylin and eosin (H&E) staining of EAT (bar = 50 μm, 20× magnification), (**b**) adipocyte area from each group, (**c**) number of crown-like structures (CLS), (**d**) transforming growth factor-β1 (TGF-β1), (**e**) interleukin 1 beta (IL-1β) level, and (**f**) chemokine (C-X-C motif) ligand 1 (CXCL-1) levels were measured. Values are displayed as box-and-whisker plots, n = 8 for individual group. Data were analyzed by two-way ANOVA followed by Tukey’s multiple comparisons test to determine the interactions or the main effects (diet and LPS stimulation). Asterisk indicates a significant main effect for LPS (*** *p* < 0.001, **** *p* < 0.0001). Pound indicates a significant main effect for diet (^##^ *p* < 0.01). Labeled means without a common letter differ significantly, *p* < 0.05. The mean values are indicated by “+” signs. HFD, high-fat diet; HFD + KO, high-fat diet + krill oil; HFD + CO, high-fat diet + coconut oil; PBS, phosphate-buffered saline; LPS, lipopolysaccharide.

To demonstrate the effect of fat replacement with KO or CO and LPS challenge on hypertrophy and macrophage infiltration in EAT, we performed H&E staining and measured fibrosis markers and expression of proinflammatory cytokines. Histological examination of EAT sections (Figure 8a) and subsequent quantification of adipocyte size confirmed very large adipocytes in rats fed with HFD (*p* < 0.001, Figure 8b). Notably, rats fed with HFD + KO exhibited much lower adipocyte hypertrophy than those fed with HFD (Figure 8a,b). Additionally, the adipocyte size of the HFD + CO group decreased by 40% after LPS treatment. The CLS is a histological feature found in the obese adipose tissue and has been demonstrated to be a proinflammatory macrophage surrounding adipocytes. The CLS was prominently observed in LPS-challenged rats (*p* < 0.01; Figure 8c). The number of CLS in rats showed a significant interaction between LPS and diet (*p* < 0.01), as it was highest in LPS-treated rats fed with HFD + CO, whereas there was no statistical significance in other experimental groups. These results indicated that fat replacement with KO or CO effectively improved LPS challenge-mediated fat inflammation, and KO responded more effectively. In both the presence and absence of LPS, TGF-β1 expression in KO-fed rats is similar to that in the HFD group; however, the HFD + CO group had significantly higher TGF-β1 expression than the other experimental groups (*p* < 0.01, Figure 8d). The expression of IL-1β and CXCL-1 in the EAT is significantly upregulated by LPS stimulation (Figure 8e,f) by 22.98-fold and 20.59-fold, respectively, compared to the PBS group (*p* < 0.0001; IL-1β, *p* < 0.001; CXCL-1). Following LPS treatment, CXCL-1 expression in the EAT of rats fed with HFD + KO or HFD + CO diet was lower than that in the HFD group. Furthermore, in the EAT of rats fed with HFD + KO with LPS treatment, IL-1β expression was lower than that in the HFD group.

## 4. Discussion

High dietary fat induces overconsumption, hepatic fat accumulation, and increased BW gain, which can further lead to obesity [57]. It has been widely demonstrated that obesity leads to a number of health complications, including diabetes, heart disease, hypertension, chronic kidney disease, and cancers [58]. The total dietary fat intake has been previously believed to be a main predictor of obesity, as fat is the most energy-dense macronutrient; however, the current focus has shifted to the quality or types of dietary fat [59]. It is well-established that a high intake of SFAs induces local inflammation and adverse cardiometabolic effects [60,61]. However, the administration of PUFAs has been reported to exert various health benefits, including potent mediators of adiposity, diet-induced inflammation, dyslipidemia, and insulin resistance in rats [62,63]. Furthermore, MCFAs have been shown to be associated with anti-inflammatory activities [64] and cardioprotective effects by raising high-density lipoproteins [65] and reducing blood pressure and heart rate [66]. Therefore, replacing SFAs with alternative ingredients to prevent obesity-related metabolic disturbances and its related risks for CVD has gained substantial attention [67,68]. Recent studies suggest that diet-induced obesity (DIO) in rats alters cytokine production and impairs liver function and morphology in response to acute inflammatory stimuli such as LPS. Despite their beneficial pharmacological activities, anti-inflammatory and cardioprotective effects of MCFAs and PUFAs in rats in response to LPS remain to be elucidated. Our investigation was designed to understand the effects of partial replacement of dietary fat with different fatty acids (*n*-3 PUFAs or MUFAs) without energy reduction from fat. We thus utilized a rat model of HFD-induced obesity to assess the impact of different fatty acids on lipid metabolism when challenged with LPS.

The HFD, often referred to as the “Western diet” contains approximately 45% of energy from fat, 36% from carbohydrates, and 19% from protein, whereas a standard chow diet is composed of 18%, 58%, and 24% of calories from fat, carbohydrates, and protein, respectively [69]. In this study, the HFD created for this study was composed of various amounts of Western blend (~83% of energy from milk fat) for the replacement of dietary fat with KO or CO. [70]. Dietary fat compositions can alter metabolic function and influence BW, composition, and whole-body energetics [71]. As summarized in Table 6, the potential of KO against obesity in experimental rats has been investigated in many studies. In C57BL/6 mice fed with a HFD, Tandy et al. [34] investigated the dose-dependent effects of dietary KO supplementation on cardiovascular and metabolic parameters and suggested that supplementation with a high dose of KO (2.5% or 5.0%) was a more beneficial effect than a low dose of KO (1.25 wt.%). Furthermore, Sun et al. [33] demonstrated that dyslipidemia, fatty liver, and glucose metabolism were improved in C57BL/6 J mice fed with HFD and with 5.0% substituent of KO for 12 weeks. Other studies that fed a HFD confirmed that supplementation with 2.0–2.5% (*w*/*w*) of KO had an anti-obesity effect. Famurewa et al. [72] and Hima et al. [73] reported that supplementation of 4~16% coconut oil in the diet improved oxidative stress, inflammation, and apoptosis via improvement in antioxidant mechanism by increasing antioxidative enzymatic activities and inflammatory signaling pathways as a post-translational manner (Table 7). 

Many studies have reported the weight-lowering effects of MCFAs [76,79,80] or PUFAs [28,81]. However, in the present study, although rats fed with HFD + KO and HFD + CO showed slightly, but significantly, lower daily food intake, we did not observe differences in BW and FER among the different groups in our experimental settings. Thus, it is likely that one cause of the discrepancies might be attributed to the insufficient quantity of the KO or CO to exhibit pronounced BW loss.

Obesity is a leading risk factor for T2D and metabolic syndrome. Chronic overfeeding triggers inflammation and further leads to peripheral insulin resistance and hyperinsulinemia [49,82]. Based on our findings from OGTT and ITT, HFD + KO and HFD + CO did not alter glucose metabolism. The average serum glucose in male SD rats at 17 weeks of age or older ranges from 106 to 184 mg/dL [83]. This indicates that HFD markedly elevated fasting glucose levels along with serum insulin, whereas HFD + KO and HFD + CO reduced serum glucose levels close to normal levels in the absence of LPS. These results indicate that both HFD + KO and HFD + CO promoted a reduction in HOMA-IR levels. HOMA-IR is a well-established method for estimating beta cell function and insulin resistance at a steady state, in which high HOMA-IR values indicate low insulin sensitivity, such as insulin resistance [84,85]. Consistent with our findings, previous studies on *n*-3 PUFAs have demonstrated its protective effects against insulin resistance [28,86]. In the present study, CO consumption in rats (without LPS administration) reduced serum glucose and insulin levels, as well as HOMA-IR values. However, the effects of MCFAs on glucose metabolism remain contradictory, with some studies observing the ameliorating effects of MCFAs on insulin resistance [87,88], whereas others do not [75,77]. Thus, further research is needed to understand the mechanisms underlying the effects of MCFAs on insulin sensitivity. Moreover, the findings of the current study indicate that LPS injection increased serum glucose and insulin levels. This is consistent with previous studies suggesting that LPS might be involved in glucose-induced hyperinsulinemia, possibly via activation of toll-like receptor 4 (TLR4) [89].

Atherogenic dyslipidemia is characterized by elevated levels of TG, TC, and LDL-C, along with reduced levels of HDL-C [90]. Studies have reported that LPS injection induces hyperlipidemia by significantly increasing total lipids, TC, TG, AST, and ALT activities [91,92,93]. A study by Khan et al. [76] showed elevation in serum TC, non-HDL-C, LDL-C, and TG of LPS-treated male SD rats. Our findings were in accordance with previous studies showing that administration of LPS promoted hyperlipidemia, accompanied by elevated TG, TC, HDL-C, non-HDL-C, and CVD expectancy markers (i.e., AC and CRF) [93,94]. Our results highlighted in Figure 3 indicate that KO and CO consumption attenuated these significantly elevated levels. These results were consistent with the report by Sun et al. that KO feeding lowered serum TC and LDL-C levels in HFD-induced dyslipidemia mice [33]. In accordance with this, several studies have reported the preventive effects of CO against dyslipidemia, associated with reduced TC, TG, phospholipids, LDL-C, and VLDL-C [95,96]. Consequently, these studies suggest that KO and CO supplementation exhibit beneficial effects, particularly on lipid metabolism and CVD expectancy. Furthermore, the AST and ALT levels are indicators of dyslipidemia and liver injury [97]. As presented in the present study, the administration of LPS induced elevated levels of serum AST and ALP activities, indicating liver damage. In accordance with our results, other studies have reported a reduction in the serum AST and ALT levels due to MCFAs [98] and PUFAs [99]. PUFAs consumption may prevent NAFLD by attenuation of ectopic fat accumulation in the liver [100,101]. Less ectopic fat accumulation could decrease hepatic damage per 2-hit hypothesis by reducing inflammation and oxidative stress [102].

Adipose tissue accumulation in obesity increases the number of macrophages and promotes local and systemic inflammation [103]. As visceral obesity is an important risk factor for developing metabolic syndrome and CVD, we assessed the weights of several visceral fat depositions, including EAT, MAT, RAT, and PAT. Our findings in Figure 2 suggest that neither KO nor CO-replaced diets showed significant effects on the weights in the liver and various fat depots. The rats fed with HFD + KO exhibited a decreasing trend in the weights of different adipose depots, in which WAT and RAT showed a diet main effect. These findings were further explored in EAT, a metabolically active tissue widely used to study lipid metabolism in rodents [104]. WAT expansion involves two distinct processes: hyperplasia and hypertrophy; however, visceral WAT expansion mainly occurs due to hypertrophy [105]. Evidence suggests that adipocyte hypertrophy is associated with increased inflammation [106,107]. WAT dysfunction is characterized by adipocyte hypertrophy, macrophage infiltration, the appearance of CLS, and elevated expression of inflammatory genes [105,108]. In accordance with the weights of various fat pads (Figure 2b–f), KO consumption in rats alleviated the development of hypertrophic EAT. However, in the present study, HFD + KO did not noticeably influence the number of CLS, implying that the KO quantity was insufficient to alter macrophage infiltration caused by HFD. These results are consistent with those of previous studies using HFD supplemented with KO [109] or fish oil [110]. Conversely, previous studies also reported adipocyte hypertrophy associated with CO, which is also indicated in our study [111,112]. HFD-induced obesity results in chronic low-grade inflammation. Thus, the effects of KO and CO on the cytokines and chemokines, such as IL-1β, MCP-1, and CXCL-1, are further assessed in the serum (Figure 5a,b), liver (Figure 6d,e), and EAT (Figure 8e,f). However, no prominent differences were noted among the different diets. Moreover, LPS exposure from Gram-negative bacterial infections induces the excessive release of proinflammatory cytokines [113,114,115]. Mechanistically, LPS binds to LPS binding protein in the blood and recruits CD14 to form a complex. After ligand binding, the complex is translocated to the TLR4 receptor complex for dimerization. The conformational change then initiates the subsequent activation of downstream inflammatory signaling through the activation of NF-κB and MAPK signaling pathways [89,98,116,117]. In the present study, after LPS treatment, the HFD + KO and HFD + CO groups showed downregulated mRNA expression of proinflammatory cytokines in the liver (CXCL-1) and EAT, such as IL-1β and CXCL-1, as compared to the HFD group. In summary, our findings on proinflammatory cytokine and chemokine levels in the serum, liver, and EAT indicate that KO and CO contents were not able to prominently downregulate the stimulation of TLR4 signaling pathway, possibly due to insufficient KO and CO levels.

Obesity is associated with various metabolic disorders, and NAFLD is one such condition. NAFLD is a chronic liver disease that ranges from benign steatosis to nonalcoholic steatohepatitis (NASH) [118]. NASH is caused by fat accumulation in the hepatocytes and may progress to fibrosis, cirrhosis, liver failure, or hepatocellular carcinoma [119]. In addition to obesity, bacterial endotoxins such as LPS are key factors in the pathogenesis of NASH [120]. The underlying mechanism of NAFLD pathogenesis is widely accepted as the “two-hit hypothesis” [121]. The onset of disease starts with the first hit involving hepatic TG accumulation and insulin resistance, followed by the second hit, resulting in more severe liver injury associated with impaired inflammatory cytokines, adipokines, mitochondrial dysfunction, and oxidative stress [121,122]. Thus, we analyzed common parameters used for the diagnosis of NAFLD to observe the hepatoprotective effects of MCFAs and PUFAs. A previous study reported that PUFAs mediate hepatic enzymes involved in anabolic pathways, including ACC and FAS; thus, promoting a decrease in hepatic adiposity [123]. In this study, although the weight of the liver was not significantly changed, hepatic TGs and lipogenesis-related genes (FAS, G6PD, and ACC) showed a decreasing pattern followed by KO consumption in the absence of LPS. A similar pattern was observed in hepatic histology and fibrosis markers according to KO supplementation. However, among the various steatosis-related parameters, CO supplementation only showed a slight improvement in G6PD compared to HFD. Our findings were consistent with previous results reported by Ferramosca et al., where supplementation of KO in the HFD prevented TG accumulation in the liver as well as de novo fatty acid synthesis in the liver [28]. These protective effects of KO against liver injury might be related to the inactivation of NF-κB/MAPK signaling pathways, as discussed above. LPS causes anabolic lipid accumulation in the liver; however, based on our study, LPS administration decreased lipid accumulation in the liver. These results might be because LPS induces strong autophagy in the liver and hepatocytes. Studies have reported that LPS-treated mice showed reduced hepatic lipid accumulation with higher induction of autophagy compared to controls [124]. However, chloroquine treatment inhibited LPS-induced autophagy and increased lipid accumulation in the liver. In this study, hepatic lipid accumulation increased in the CO diet in LPS-treated rats. Furthermore, the expression of adipogenesis transcription factors and adipogenic enzymes increased slightly in KO- and CO-fed rats. This indicates that an increase in endotoxin-induced autophagy may modulate lipid metabolism changes, and further studies are needed to identify the underlying mechanisms. These findings indicate that partial replacement of HFD with KO might be partly associated with the prevention of liver steatosis and fibrosis.

## 5. Conclusions

Diet-induced obesity leads to various adverse health complications, such as hyperglycemia, dyslipidemia, and hypertension, and further increases the risk of CVD. Taken together, we have shown that partial replacement of HFD with KO or CO improved DIO-induced adiposity, glucose homeostasis, insulin resistance, serum lipid profiles, and hepatic steatosis. More pronounced results were observed in the rats that consumed KO. The protective effects of KO against liver injury might be associated with the inactivation of NF-κB/MAPK signaling pathways. These results suggest that *n*-3 PUFAs or MCFAs supplementation may be utilized as a replacement source of a HFD to ameliorate the metabolic dysregulation caused by obesity. However, long-term studies are needed to thoroughly understand the mechanisms underlying the utilization of *n*-3 PUFAs or MCFAs supplementation in clinical trials.

## Figures and Tables

**Table 1:** Composition of the experimental diets.

	Groups	HFD	HFD + KO	HFD + CO
Ingredient (g/kg)				
Casein		220	220	220
L-cysteine		3.4	3.4	3.4
Sucrose		100	100	100
Corn starch		160	160	160
Dextrose		155	155	155
Cellulose		58	58	58
Mineral mix ^1^		43	43	43
Vitamin mix ^2^		19	19	19
Choline bitartrate		2.8	2.8	2.8
Western blend ^3^		184	134	134
Lard		55	55	55
*tert*-Butylhydroquinone		0.034	0.034	0.034
Krill oil			50	
Coconut oil				50
Energy (kcal/g)		4.78	4.78	4.78
Fat (kcal %)		45%	45%	45%

^1^ AIN-93-GX, ^2^ AIN-93-VX, ^3^ Western blend consisting of milk fat, moisture, and milk solid non-fat in a ratio of 82.9:15.7:1.4. Abbreviations: HFD, high-fat diet; HFD + KO, high-fat diet + krill oil; HFD + CO, high-fat diet + coconut oil.

**Table 2:** Fatty acid composition of the experimental diets.

	Groups	HFD	HFD + KO	HFD + CO
Fatty Acid (% Total Fatty Acids)				
C_4:0_, Butyric acid		0.49 ± 0.03 ^a^	0.36 ± 0.02 ^b^	0.35 ± 0.02 ^b^
C_6:0_, Caproic acid		2.08 ± 0.11 ^a^	1.63 ± 0.09 ^b^	1.69 ± 0.09 ^b^
C_8:0_, Caprylic acid		1.98 ± 0.10 ^b^	1.67 ± 0.08 ^b^	4.37 ± 0.22 ^a^
C_10:0_, Capric acid		4.10 ± 0.21 ^b^	3.19 ± 0.16 ^c^	4.88 ± 0.25 ^a^
C_12:0_, Lauric acid		6.20 ± 0.31 ^b^	4.65 ± 0.23 ^c^	15.91 ± 0.80 ^a^
C_13:0_, Tridecanoic acid		ND	ND	ND
C_14:0_, Myristic acid		11.53 ± 0.58 ^a^	9.83 ± 0.50 ^b^	12.04 ± 0.60 ^a^
C_14:1_, Myristoleic acid		0.76 ± 0.04 ^b^	1.00 ± 0.05 ^a^	0.76 ± 0.04 ^b^
C_15:0_, Pentadecanoic acid		1.88 ± 0.10 ^a^	1.53 ± 0.08 ^b^	1.26 ± 0.06 ^c^
C_16:0_, Palmitic acid		24.79 ± 1.24 ^a^	21.97 ± 1.10 ^b^	18.96 ± 0.95 ^c^
C_16:1_, Palmitoleic acid		2.5 ± 0.13 ^b^	3.78 ± 0.19 ^a^	2.05 ± 0.11 ^c^
C_17:0_, Heptadecanoic acid		2.98 ± 0.15 ^b^	4.31 ± 0.22 ^a^	3.08 ± 0.16 ^b^
C_18:0_, Stearic acid		13.5 ± 0.68 ^a^	10.99 ± 0.55 ^b^	11.13 ± 0.56 ^b^
C_18:1_, Oleic acid		18.23 ± 0.92	16.64 ± 0.83	16.32 ± 0.82
C_18:2_, Linoleic acid		6.63 ± 0.33	5.95 ± 0.30	6.25 ± 0.32
C_18:3_, Linolenic acid		1.27 ± 0.07 ^b^	1.68 ± 0.08 ^a^	ND
C_20:1_, Eicosenoic acid		0.36 ± 0.02 ^b^	0.48 ± 0.02 ^a^	0.37 ± 0.02 ^b^
C_20:2_, Eicosadienoic acid		0.29 ± 0.02 ^b^	0.96 ± 0.05 ^a^	0.27 ± 0.02 ^b^
C_20:3_, Eicosatienoic acid		ND	0.50 ± 0.03 ^a^	ND
C_20:5_, Eicosapentaenoic acid		0.22 ± 0.01 ^b^	5.41 ± 0.28 ^a^	0.13 ± 0.01 ^b^
C_22:1_, Erucic acid		ND	0.30 ± 0.02 ^a^	ND
C_22:6_, Docosahexaenoic acid		ND	3.17 ± 0.16 ^a^	ND
Others ^1^		0.20 ± 0.01 ^a^	ND	0.18 ± 0.01 ^b^
SFAs		69.55 ± 3.48 ^a^	60.14 ± 3.01 ^b^	73.67 ± 3.69 ^a^
MUFAs		21.85 ± 1.10 ^ab^	22.20 ± 1.11 ^a^	19.50 ± 0.98 ^b^
PUFAs		8.41 ± 0.42 ^b^	17.66 ± 0.89 ^a^	6.65 ± 0.34 ^c^
*n*-6		6.92 ± 0.35	6.91 ± 0.35	6.52 ± 0.33
*n*-3		1.50 ± 0.08 ^b^	10.75 ± 0.54 ^a^	0.13 ± 0.01 ^c^

^1^ Others; trans-C_18:1_, trans-C_18:2_. Values are expressed as mean ± standard deviation (n = 3 per group). Values with different letters are significantly different using the Tukey’s multiple comparison post hoc test at *p* < 0.05. Abbreviations: HFD, high-fat diet; HFD + KO, high-fat diet + krill oil; HFD + CO, high-fat diet + coconut oil; SFAs, saturated fatty acids; MUFAs, monounsaturated fatty acids; PUFAs, polyunsaturated fatty acids; ND, not detected.

**Table 3:** Primer sequences for quantitative real-time polymerase chain reaction (qRT-PCR).

Gene		Primer Sequence
Il-1β	Forward	5′-AAA AAT GCC TCG TGC TGT CT-3′
	Reverse	5′-TCG TTG CTT GTC TCT CCT TG-3′
CXCL-1	Forward	5′-CCA CAC TCA AGA ATG GTC GC-3′
	Reverse	5′-GTT GTC AGA AGC CAG CGT TC-3′
Fas	Forward	5′-AAA AGG AAA GTA GAG TGT GC-3′
	Reverse	5′-GAC ACA TTC TGT TCA CTA CAG-3′
G6pd	Forward	5′-GTT TGG CAG CGG CAA CTA A-3′
	Reverse	5′-GGC ATC ACC CTG GTA CAA CTC-3′
C/EBP-α	Forward	5′-GCC AAG AAG TCG GTG GAT AA-3′
	Reverse	5′-CGG TCA TTG TCA CTG GTC AA-3′
Scd1	Forward	5′-TGT TCG TCA GCA CCT TCT TG-3′
	Reverse	5′-AGT TGA TGT GCC AGC GGT A-3′
Acc	Forward	5′-CAA CGC CTT CAC ACC ACC TT-3′
	Reverse	5′-AGC CCA TTA CTT CAT CAA AGA TCC T-3′
Tgf-β1	Forward	5′-ACC GAC CCT TCC TGC TCC TCA T-3′
	Reverse	5′-GAT CCA CTT CCA ACC CAG GTC CT-3′
Gapdh	Forward	5′-CTG TGT CTT TCC GCT GTT TTC-3′
	Reverse	5′-TGT GCT GTG CTT ATG GTC TCA-3′

Abbreviations: Il-1β, interleukin-1β; CXCL-1, C-X-C motif chemokine ligand 1; Fas, fatty acid synthesis; G6pd, glucose-6-phosphate dehydrogenase; C/EBP-α, CCAAT enhancer-binding protein α; Scd-1, stearoyl-CoA desaturase-1; Acc, acetyl-CoA carboxylase; Tgf-beta 1, transforming growth factor beta 1; Gapdh, glyceraldehyde 3-phosphate dehydrogenase.

**Table 4:** Statistical results of the two-way ANOVA for the effect of LPS and diet and LPS X diet interaction.

Parameter	Factor, *p* Values		
	LPS	Diet	LPS X Diet
Relative liver and adipose tissue weights			
Liver	** *p* < 0.01	ns *p* = 0.931	ns *p* = 0.057
WAT	ns *p* = 0.832	* *p* < 0.05	ns *p* = 0.884
EAT	ns *p* = 0.509	ns *p* = 0.079	ns *p* = 0.926
MAT	ns *p* = 0.555	ns *p* = 0.265	ns *p* = 0.673
RAT	ns *p* = 0.995	** *p* < 0.01	ns *p* = 0.508
PAT	ns *p* = 0.485	ns *p* = 0.134	ns *p* = 0.925
Serum lipid levels			
Triglyceride	** *p* < 0.01	**** *p* < 0.0001	**** *p* < 0.0001
Total cholesterol	**** *p* < 0.0001	* *p* < 0.05	ns *p* = 0.589
HDL-cholesterol	** *p* < 0.01	ns *p* = 0.095	ns *p* = 0.227
Non-HDL-cholesterol	**** *p* < 0.0001	* *p* < 0.05	ns *p* = 0.735
Atherogenic coefficient	**** *p* < 0.0001	*** *p* < 0.001	ns *p* = 0.997
Cardiac risk factor	**** *p* < 0.0001	*** *p* < 0.001	ns *p* = 0.997
Serum glucose and insulin levels			
Glucose	ns *p* = 0.782	* *p* < 0.05	*** *p* < 0.001
Insulin	* *p* < 0.05	* *p* < 0.05	* *p* < 0.05
HOMA-IR	ns *p* = 0.078	* *p* < 0.05	*** *p* < 0.001
Fatty acid levels in whole blood			
C_14:0_, Myristic acid	ns *p* = 0.428	ns *p* = 0.091	ns *p* = 0.658
C_16:0_, Palmitic acid	ns *p* = 0.199	ns *p* = 0.767	ns *p* = 0.28
C_16:1_, Palmitoleic acid	ns *p* = 0.311	ns *p* = 0.196	ns *p* = 0.397
C_18:0_, Stearic acid	ns *p* = 0.643	* *p* < 0.05	ns *p* = 0.35
C_18:1_, Oleic acid	ns *p* = 0.26	ns *p* = 0.055	ns *p* = 0.353
C_18:2_, Linoleic acid	ns *p* = 0.908	* *p* < 0.05	ns *p* = 0.105
C_18:3*n*-3_, α-linolenic acid	ns *p* = 0.499	ns *p* = 0.336	ns *p* = 0.636
C_18:3*n*-6_, γ-linolenic acid	ns *p* = 0.276	ns *p* = 0.287	ns *p* = 0.195
C_20:1_, Eicosenoic acid	ns *p* = 0.885	ns *p* = 0.155	ns *p* = 0.700
C_20:2_, Eicosadienoic acid	ns *p* = 0.274	ns *p* = 0.23	ns *p* = 0.402
C_20:3_, Dihomo-γ-linolenic acid	ns *p* = 0.28	ns *p* = 0.817	* *p* < 0.05
C_20:4_, Aachidonic acid	ns *p* = 0.941	*** *p* < 0.001	ns *p* = 0.111
C_20:5_, Eicosapentaenoic acid	* *p* < 0.05	*** *p* < 0.001	* *p* < 0.05
C_22:4_, Docosatetraenoic acid	ns *p* = 0.335	*** *p* < 0.001	ns *p* = 0.405
C_22:5_, Docosapentaenoic acid	ns *p* = 0.979	*** *p* < 0.001	ns *p* = 0.67
C_22:6*n*-3_, Docosahexaenoic acid	* *p* < 0.05	*** *p* < 0.001	ns *p* = 0.417
C_22:6*n*-6_, Docosapentaenoic acid	ns *p* = 0.716	*** *p* < 0.001	ns *p* = 0.288
C_24:0_, Lignoceric acid	ns *p* = 0.504	ns *p* = 0.525	ns *p* = 0.899
C_24:1_, Nervonic acid	ns *p* = 0.335	* *p* < 0.05	ns *p* = 0.552
Others	ns *p* = 0.743	** *p* < 0.01	ns *p* = 0.29
SFAs	ns *p* = 0.169	ns *p* = 0.875	ns *p* = 0.269
MUFAs	ns *p* = 0.402	ns *p* = 0.057	ns *p* = 0.356
PUFAs	ns *p* = 0.877	* *p* < 0.05	ns *p* = 0.464
*n*-6	ns *p* = 0.904	*** *p* < 0.001	ns *p* = 0.404
*n*-3	ns *p* = 0.609	*** *p* < 0.001	ns *p* = 0.891
*n*-6/*n*-3	ns *p* = 0.385	*** *p* < 0.001	ns *p* = 0.780
AA/EPA	ns *p* = 0.879	*** *p* < 0.001	ns *p* = 0.993
Serum cytokine levels			
IL-1β	ns *p* = 0.219	* *p* < 0.05	* *p* < 0.05
MCP-1	**** *p* < 0.0001	ns *p* = 0.056	ns *p* = 0.338
Serum levels of hepatic function parameter			
AST	**** *p* < 0.0001	**** *p* < 0.0001	* *p* < 0.05
ALT	**** *p* < 0.0001	** *p* < 0.01	ns *p* = 0.357
ALP	* *p* < 0.05	ns *p* = 0.958	ns *p* = 0.282
Hepatic histology, inflammatory and fibrosis related gene expressions			
Hepatic histological score	* *p* < 0.05	** *p* < 0.01	ns *p* = 0.357
TGF-β1 mRNA expression	* *p* < 0.05	* *p* < 0.05	ns *p* = 0.713
IL-1β mRNA expression	**** *p* < 0.0001	ns *p* = 0.532	ns *p* = 0.461
CXCL-1 mRNA expression	*** *p* < 0.001	ns *p* = 0.263	ns *p* = 0.368
Hepatic lipid level, lipogenesis related gene expressions			
Hepatic triglyceride	**** *p* < 0.0001	* *p* < 0.05	ns *p* = 0.733
Hepatic total cholesterol	**** *p* < 0.0001	ns *p* = 0.168	ns *p* = 0.666
FAS mRNA expression	ns *p* = 0.945	ns *p* = 0.466	ns *p* = 0.058
G6PD mRNA expression	* *p* < 0.05	**** *p* < 0.0001	**** *p* < 0.0001
ACC mRNA expression	*** *p* < 0.001	ns *p* = 0.062	ns *p* = 0.341
C/EBP-α mRNA expression	**** *p* < 0.0001	ns *p* = 0.132	** *p* < 0.01
SCD1 mRNA expression	**** *p* < 0.0001	** *p* < 0.01	** *p* < 0.01
EAT inflammatory and fibrosis related gene expressions			
Adipocyte area	ns *p* = 0.157	*** *p* < 0.001	** *p* < 0.01
Number of CLS	** *p* < 0.01	ns *p* = 0.294	** *p* < 0.01
IL-1β mRNA expression	**** *p* < 0.0001	ns *p* = 0.485	ns *p* = 0.471
CXCL-1 mRNA expression	*** *p* < 0.001	ns *p* = 0.221	ns *p* = 0.222
TGF-β1 mRNA expression	**** *p* < 0.0001	** *p* < 0.01	ns *p* = 0.302

Ns = not significant; * *p* < 0.05, ** *p* < 0.01, *** *p* < 0.001, **** *p* < 0.0001. Abbreviations: WAT, white adipose tissue; EAT, epididymal adipose tissue; MAT, mesenteric adipose tissue; RAT, retroperitoneal adipose tissue; PAT, perirenal adipose tissue; HDL, high-density lipoprotein; HOMO-IR, homeostatic model assessment of insulin resistance; SFAs, saturated fatty acids; MUFAs, monounsaturated fatty acids; PUFAs, polyunsaturated fatty acids; AA, arachidonic acid; EPA, eicosapentaenoic acid; Acc, acetyl-CoA carboxylase; C/EBP-α, CCAAT enhancer-binding protein α; CXCL-1, C-X-C motif chemokine ligand 1; ALP, alkaline phosphatase; ALT, alanine aminotransferase; AST, aspartate transaminase; Fas, fatty acid synthesis; G6pd, glucose-6-phosphate dehydrogenase; Il-1β, interleukin-1 beta; MCP-1, monocyte chemoattractant protein-1; Scd-1, stearoyl-CoA desaturase-1; Tgf-β 1, transforming growth factor beta 1; CLS, crown-like structures.

**Table 5:** Effects of partial replacement of dietary fat with krill oil or coconut oil on the fatty acid composition of whole blood in rats after the LPS challenge.

	Groups	PBS			LPS		
Fatty Acid (% Total Fatty Acids)		HFD	HFD + KO	HFD + CO	HFD	HFD + KO	HFD + CO
C_14:0_, Myristic acid		1.11 ± 0.45	0.83 ± 0.16	1.53 ± 0.59	0.88 ± 0.27	0.92 ± 0.19	1.24 ± 0.42
C_16:0_, Palmitic acid		28.47 ± 0.55	30.5 ± 0.52	26.9 ± 0.95	28.07 ± 0.49	22.43 ± 11.12	26.57 ± 0.21
C_16:1_, Palmitoleic acid		1.17 ± 0.4	0.74 ± 0.27	0.80 ± 0.39	0.84 ± 0.04	0.85 ± 0.25	0.61 ± 0.09
C_18:0_, Stearic acid		15.17 ± 1.1	16.57 ± 0.87	16.97 ± 0.64	15.77 ± 0.68	15.83 ± 0.23	16.57 ± 0.95
C_18:1_, Oleic acid		18.03 ± 3.40	14.67 ± 1.45	13.93 ± 2.03	17.37 ± 1.05	15.9 ± 0.20	16.4 ± 0.92
C_18:2_, Linoleic acid		7.70 ± 1.08	7.92 ± 0.50	7.82 ± 0.63	6.66 ± 0.64	8.39 ± 0.73	8.51 ± 0.43
C_18:3*n*-3_, α-linolenic acid		0.15 ± 0.11	0.06 ± 0.05	0.10 ± 0.06	0.11 ± 0.12	0.09 ± 0.03	0.05 ± 0.02
C_18:3*n*-6_, γ-linolenic acid		0.05 ± 0.02	0.03 ± 0.02	0.08 ± 0.05	0.04 ± 0.02	0.04 ± 0.01	0.04 ± 0.01
C_20:1_, Eicosenoic acid		0.18 ± 0.01	0.10 ± 0.06	0.12 ± 0.04	0.16 ± 0.12	0.09 ± 0.06	0.16 ± 0.02
C_20:2_, Eicosadienoic acid		0.32 ± 0.12	0.18 ± 0.07	0.26 ± 0.08	0.20 ± 0.07	0.18 ± 0.06	0.24 ± 0.07
C_20:3_, Dihomo-γ-linolenic acid		0.65 ± 0.07 ^a^	0.55 ± 0.08 ^ab^	0.58 ± 0.11 ^ab^	0.50 ± 0.05 ^b^	0.63 ± 0.04 ^a^	0.55 ± 0.04 ^ab^
C_20:4_, Aachidonic acid		20.00 ± 2.25	11.00 ± 0.92	23.7 ± 1.06	21.73 ± 1.19	11.00 ± 0.20	22.10 ± 0.95
C_20:5_, Eicosapentaenoic acid		0.36 ± 0.12 ^c^	7.58 ± 0.48^a^	0.22 ± 0.06 ^c^	0.39 ± 0.12 ^c^	6.64 ± 0.48 ^b^	0.20 ± 0.03 ^c^
C_22:4_, Docosatetraenoic acid		0.89 ± 0.24	0.09 ± 0.06	1.57 ± 0.37	0.89 ± 0.07	0.08 ± 0.02	1.3 ± 0.11
C_22:5_, Docosapentaenoic acid		1.27 ± 0.29	3.47 ± 0.64	1.22 ± 0.17	1.2 ± 0.46	3.67 ± 0.1	1.07 ± 0.14
C_22:6*n*-3_, Docosahexaenoic acid		2.57 ± 0.41	3.97 ± 0.74	2.11 ± 0.25	3.1 ± 0.42	4.89 ± 0.51	2.31 ± 0.21
C_22:6*n*-6_, Docosapentaenoic acid		0.24 ± 0.07	0.02 ± 0.01	0.66 ± 0.18	0.35 ± 0.06	0.06 ± 0.01	0.57 ± 0.17
C_24:0_, Lignoceric acid		0.32 ± 0.09	0.36 ± 0.23	0.37 ± 0.04	0.26 ± 0.04	0.35 ± 0.05	0.33 ± 0.05
C_24:1_, Nervonic acid		0.21 ± 0.05	0.29 ± 0.09	0.11 ± 0.03	0.12 ± 0.11	0.23 ± 0.15	0.13 ± 0.03
Others ^1^		0.28 ± 0.02	0.20 ± 0.05	0.21 ± 0.04	0.23 ± 0.05	0.16 ± 0.02	0.16 ± 0.04
SFAs		45.07 ± 1.27	48.26 ± 1.57	45.76 ± 1.1	44.97 ± 0.68	39.54 ± 11.38	44.7 ± 1.29
MUFAs		19.6 ± 3.76	15.79 ± 1.67	14.96 ± 2.37	18.48 ± 1.08	17.07 ± 0.53	17.3 ± 1.01
PUFAs		34.2 ± 2.79	34.86 ± 2.29	38.31 ± 1.63	35.17 ± 1.26	35.68 ± 0.84	36.92 ± 1.15
*n*-6		29.85 ± 2.42	19.79 ± 0.63	34.66 ± 1.69	30.37 ± 0.51	20.38 ± 0.68	33.3 ± 1.23
*n*-3		4.35 ± 0.45	15.07 ± 1.67	3.65 ± 0.15	4.8 ± 0.75	15.29 ± 0.94	3.62 ± 0.08
*n*-6/*n*-3		6.88 ± 0.48	1.32 ± 0.11	9.51 ± 0.72	6.43 ± 0.98	1.34 ± 0.11	9.21 ± 0.53
AA/EPA		58.24 ± 12.03	1.45 ± 0.06	112.14 ± 27.47	59.25 ± 17.89	1.66 ± 0.15	114.63 ± 21.89

^1^ Others; trans-C_16:1_, trans-C_18:1_, trans-C_18:2_. Values are presented as the mean ± standard deviation (n = 3 per group). Data are analyzed by two-way ANOVA followed by Tukey’s multiple comparisons test to determine the interactions or the main effects (diet and LPS stimulation). Abbreviations: HFD, high-fat diet; HFD + KO, high-fat diet + krill oil; HFD + CO, high-fat diet + coconut oil; PBS, phosphate-buffered saline; LPS, lipopolysaccharide; SFAs, saturated fatty acids; MUFAs, monounsaturated fatty acids; PUFAs, polyunsaturated fatty acids; AA, arachidonic acid; EPA, eicosapentaenoic acid.

**Table 6:** Effects of krill oil in animal models with metabolic disease.

Strain	Inducer	Treatment	Biological Markers	Ref.
C57BL/6J mouse	HFD	5 % (*w*/*w*) for 12 weeks	Liver and epididymal fat weights ↓	[33]
			Serum TC, LDL-C levels ↓	
			Serum and liver MDA level, ALT activity ↓	
			Serum and liver SOD levels ↑	
C57BL/6N mouse	HFD	2% (*w*/*w*) for 12 weeks	Body weight and abdominal fat (visceral and subcutaneous) contents ↓	[22]
			Hepatic weight and hepatic steatosis ↓	
			Serum TG, LDL-C levels ↓	
			Fatty acid synthesis related genes expression ↓	
Sprague-Dawley rats	HFD	2.5% (*w*/*w*) for 12 weeks	Body weight gain ↓	[28]
			Hepatic fatty acid oxidation related gene expression ↑	
			Plasma TG levels ↓	
			Hepatic TG, TC levels ↓	
			Glucose and insulin concentration ↓	
C57BL/6 mouse	HFD	1.25, 2.5, and 5% (*w*/*w*) for 8 weeks	Liver weight ↓	[34]
			Hepatic TG, TC levels ↓	
			Serum TC, glucose ↓	
			Serum adiponectin ↑	
			Hepatic TNF-α level and mRNA expression ↓	
			Hepatic FA synthesis associated with gene expression (FAS, ACC, SCD-1) ↓	
			Hepatic FA catabolism associated with gene expression (MGLL, LIPE) ↓	
			Hepatic FA oxidation associated with gene expression (CPT-1) ↓	
			Hepatic cholesterol metabolism associated with gene expression (HMG CoA-R, LDL-R) ↓	
			Hepatic transcription factors gene expression (SREBP-1c, SREBP-2, PPAR-α) ↓	

↑, increased; ↓, decreased; HFD, high-fat diet; ACC, acetyl-CoA carboxylase; ALT, alanine transaminase; CPT-1, carnitine palmitoyl transferase I; FA, fatty acid; FAS, fatty acid synthesis; HMG-CoA; 3-hydroxy-3-methylglutaryl coenzyme A reductase; LDL-C, low-density lipoprotein-cholesterol; LDL-R, low-density lipoprotein receptor; LIPE, hormone-sensitive lipase; MDA, malondialdehyde; MGLL, monoglyceride lipase; PPAR-α, peroxisome proliferator-activated receptor-α; SCD-1, stearoyl-CoA desaturase-1; SOD, sodium oxide dismutase; SREBP, sterol regulatory element binding protein; TC, total cholesterol; TG, triglyceride; TNF-α, tumor necrosis factor-α.

**Table 7:** Effects of coconut oil in animal models with metabolic disease.

Strain	Inducer	Treatment	Biological Markers	Ref.
Wistar rats	Paracetamol, 750 mg/kg body weight (i.p.)	Virgin coconut oil (phoscoliv) 0.5 mL/100 g body weight	Serum proinflammatory cytokines (IL-6, TNF-α) ↓	[74]
			Histological scoring of liver tissue ↓	
Wistar rats	HFD (60% of calories from fat)	high-coconut oil diet (60% of calories from coconut oil) for 3 days	Serum glucose, insulin, leptin concentrations ↓	[75]
			Protein levels of inflammatory markers in the hippocampi and prefrontal cortices (IL-1β, IL-6, TNF-α and NF-κB) ↓	
Sprague-Dawley rats	LPS 10 mg/kg body weight (i.p.)	Lauric acid 50, 100 mg/kg body weight (p.o.) for 14 days	Serum AST, ALT levels ↓	[76]
			Serum TC, non-HDL-C, LDL-C and TG ↓	
			Serum HDL-C ↑	
			Serum proinflammatory cytokines (TNF-α, IL-6, IL-1β) ↓	
			Hepatic proinflammatory cytokines (TNF-α, IL-6, IL-1β) ↓	
			Hepatic protein expression of TLR4, MyD88, NF-κB, TNF-α, IL-1β ↓	
C57BL/6 mouse	HFD	1.25, 2.5, and 5% (*w*/*w*) for 8 weeks	Liver weight ↓	[77]
			Hepatic TG, TC levels ↓	
			Serum TC, glucose ↓	
			Serum adiponectin ↑	
			Hepatic TNF-α level, mRNA expression ↓	
			Hepatic FA synthesis associated with gene expression (FAS, ACC, SCD-1) ↓	
Wistar rats	Gentamicin, 100 mg/kg body weight (i.p.)	Virgin coconut oil 10% (*w*/*w*) for 16 days	Renal oxidative stress index (SOD, CAT, GPx, GSH, MDA) ↓	[78]
			Renal inflammatory cytokines (IL-1β, IL-6, TNF-α, NO, iNOS) ↓	
			Renal NF-κB level ↓	
			Renal caspase-3 activity ↓	
			Renal histomorphology ↓	
Wistar rats		Virgin coconut oil 4, 8, 16% (*w*/*w*) for 30 days	Proinflammatory cytokine (TNF-α) production in the splenocytes ↓	[73]
			p-mTOR, SIRT1, and p-LKB1 expression in the spleen ↑	
			p-ERK1/2/Total ERK1/2, p-CREB/Total CREB expression in the spleen ↑	
			Splenic SOD and CAT activities ↑	
			Serum HDL-C level ↑	

↑, increased; ↓, decreased; HFD, high-fat diet; ACC, acetyl-CoA carboxylase; ALT, alanine aminotransferase; AST, aspartate transaminase; CAT, catalase; CREB, cAMP response element-binding protein; ERK, extracellular-signal-regulated kinase; FA, fatty acid; FAS, fatty acid synthase; GPx, glutathione peroxidase; GSH, glutathione; IL-1β, interleukin-1β; IL-6, interleukin 6; iNOS, inducible nitric oxide synthase; LDL-C, low-density lipoprotein-cholesterol; MDA, malondialdehyde; mTOR, mammalian target of rapamycin; MYD88, myeloid differentiation primary response 88; NF-κB, nuclear factor kappa-light-chain-enhancer of activated B cells; non-HDL-C, non-high-density lipoprotein cholesterol; NO, nitric oxide; SCD-1, stearoyl-CoA desaturase; SOD, sodium oxide dismutase; SIRT1, sirtuin 1; LKB1, liver kinase B1; TC, total cholesterol; TG, triglyceride; TNF-α, tumor necrosis factor-α; TLR4, toll-like receptor 4.

## Data Availability

The data presented in this study are available from the corresponding authors upon request.
